# Editorial: Insights into pain mechanisms - 2024/2025

**DOI:** 10.3389/fpain.2026.1925507

**Published:** 2026-07-29

**Authors:** Dai-Qiang Liu, Ya-Qun Zhou

**Affiliations:** Department of Anesthesiology and Pain Medicine, Hubei Key Laboratory of Geriatric Anesthesia and Perioperative Brain Health, and Wuhan Clinical Research Center for Geriatric Anesthesia, Tongji Hospital, Tongji Medical College, Huazhong University of Science and Technology, Wuhan, China

**Keywords:** artificial intelligence, chronic pain, neuroinflammation, oxidative stress, pain mechanisms

Despite decades of study, pain is still one of the major challenges in both clinical and scientific terms. Chronic pain affects a substantial proportion of the adult population and is associated with considerable personal, social, and healthcare burdens (1, 2). Importantly, chronic pain is no longer regarded only as a symptom secondary to tissue injury or disease, but is considered rather as a complex condition that may involve persistent maladaptive changes in nociceptive processing, affective regulation, and behavior (3). Although substantial progress has been made in defining nociceptive processes and pain neuroanatomy, effective treatment is still limited by the heterogeneous nature and poor understanding of the mechanisms behind the onset, maintenance, modulation, and remission of pain experience. In this regard, the Research Topic “Insights into Pain Mechanisms - 2024/2025” has been created to provide recent advances in pain neurobiology, particularly focusing on molecular pathways, neuroimmune interactions, brain circuits, translational approaches, and innovative technologies that can be useful in mechanism-based and personalized pain management.

The five articles featured within this Research Topic represent the range of current research into mechanisms of pain. Together, they move across levels of analysis, from intracellular stress signaling and transcriptomic responses to descending brainstem modulation, multidimensional clinical phenotyping, and artificial intelligence (AI)-supported approaches with potential relevance to precision pain management. Such an integrative approach is especially appropriate in light of the fact that recent advances in the field of pain emphasize the importance of a multimodal approach which addresses biological mechanisms as well as brain-behavioral aspects of pain perception and disability (4). The importance of studying mechanisms of pain is not only relevant to developing drugs, but non-pharmacological interventions as well.

Alshahrani and Reddy examined the relationship between pain measures of various dimensions and balance control in community-dwelling older adults. By integrating pressure pain threshold, pain intensity, pain catastrophizing, posturographic parameters, and functional reach performance, their study showed that not only the sensory but also cognitive-affective aspects of pain are independent predictors of postural instability. Chronic pain was linked to reduced functional reach, and longer pain duration predicted greater sway area. These findings take pain mechanism studies beyond the study of sensory processing alone and highlight how persistent pain can affect motor control, risk of falls, and functional independence in older adults.

Chen et al. investigated the mechanism of electrothermal acupuncture in a model of postherpetic neuralgia in rats induced by resiniferatoxin. Electrothermal acupuncture enhanced mechanical withdrawal thresholds, reduced pro-inflammatory cytokines, inhibited reactive oxygen species production, and modulated ATP abnormalities in peripheral and spinal tissues. Moreover, the transcriptomic profiling indicated the candidate genes involved in type I interferon-mediated innate immune pathways, including Mx1, Irf7, Rtp4, Oasl2, and Ifit1bl as central genes. These findings suggest that electrothermal acupuncture may influence neuropathic pain through coordinated regulation of neuroinflammatory, oxidative, metabolic, and innate immune-related pathways.

D'Souza et al. presented a review on descending pain modulation in humans by summarizing existing primary literature data on the brain and spinal cord in pain modulation. Their review reinforces the critical involvement of the periaqueductal gray and rostral medulla, and also demonstrates that cortical and limbic regions (prefrontal, anterior cingulate, insular, and amygdala) are involved in the pain processing pathway in a context-dependent manner. Moreover, they also demonstrate that cognitive and emotional processes, such as distraction, imagery, catastrophizing, and anxiety, can modify the pathway of descending pain regulation through altered connectivity between the cortex and brainstem. These findings underscore the importance of studying human pain modulation directly and may inform the rational development of therapeutic approaches such as transcranial direct current stimulation and spinal cord stimulation.

Uckac et al. provided a comprehensive translational approach to chronic pain by incorporating genetics, brain imaging, digital phenotyping, and AI. Their review reveals chronic pain as an independent medical condition influenced by the interplay of biological, psychological, and social factors. Genetic overlaps with psychiatric disorders and the immune system, disturbances in the prefrontal, insula, and limbic regions, as well as the use of wearables and AI, are pointed out by the authors as potentially beneficial elements that will be helpful in the objective monitoring and management of patients in the future. This contribution illustrates how mechanism-informed stratification may help move pain care beyond symptom reporting toward data-driven precision medicine.

Niu et al. emphasized endoplasmic reticulum stress as a potential target for treatment of neuropathic pain. Their review provides an insight into how the chronic activation of the unfolded protein response, mediated by PERK, IRE1*α*, and ATF6 signaling, leads to neuroinflammation, oxidative stress, ferroptosis, and dysfunctional neuron-glia interaction. Importantly, they discuss the specificity of endoplasmic reticulum stress in the different types of cells, such as immune cells, sensory neurons, and oligodendrocytes. This cell-specific perspective provides a useful framework for developing targeted interventions that modulate pathological stress signaling without disrupting adaptive homeostatic responses.

Collectively, these articles demonstrate that pain mechanisms cannot be understood through a single pathway, tissue, or discipline. Rather, pain arises from the interaction between peripheral nociceptor input, immune and metabolic signals, glia and neurons, descending control pathways, cognitive affective factors, and a greater clinical context. A shared message across the Research Topic is that mechanism-based pain research is moving from isolated molecular or behavioral observations toward multilevel integration across cells, circuits, systems, and clinical phenotypes. The convergence of non-pharmacological intervention studies, human circuit-level evidence, and computational phenotyping also suggests that future analgesic development will depend increasingly on linking mechanistic biomarkers with clinically meaningful outcomes. This Research Topic also highlights a clear translational direction: future pain research should combine mechanistic depth with clinically meaningful phenotyping, longitudinal validation, and technologies capable of integrating molecular, neural, behavioral, and environmental data.

In summary, “Insights into Pain Mechanisms - 2024/2025” provides a timely overview of recent advances in the field of pain science. The articles in Research Topic help us learn more about topics such as chronic pain, descending modulation, aging-related functional consequences, and precision pain management. The combination of molecular neuroscience, human systems neurobiology, and translational intervention studies is critical in order to make pain management more effective and personalized.
